# Endovascular-assisted microsurgical clipping of ophthalmic segment aneurysms

**DOI:** 10.1007/s00701-026-06847-x

**Published:** 2026-03-26

**Authors:** Ehsan Dowlati, Seok Yoon Oh, Danielle Golub, Timothy G. White, Ahmad A. Ballout, Thomas W. Link, Athos Patsalides, Jeffrey M. Katz, Amir R. Dehdashti

**Affiliations:** 1 Department of Neurosurgery, Donald and Barbara Zucker School of Medicine at Hofstra/Northwell, North Shore University Hospital, Northwell Health, 300 Community Drive, Suite 10 Monti, Manhasset, NY 11030 USA; 2 Department of Neurology, Donald and Barbara Zucker School of Medicine at Hofstra/Northwell, Northwell Health, Manhasset, NY USA

**Keywords:** Clinoid, Intraoperative angiogram, Microsurgical clipping, Ophthalmic segment aneurysms, Temporary balloon occlusion

## Abstract

**Background:**

Proximal arterial control is critical for safe and effective microsurgical clipping of ophthalmic segment aneurysms (OSAs). Traditionally, this is achieved via neck dissection and temporary clamping of the cervical internal carotid artery (ICA). Advances in endovascular technology have introduced temporary balloon occlusion (TBO) as a potentially less invasive alternative. This study aims to assess the utility of TBO during microsurgical clipping of OSAs.

**Methods:**

A retrospective review was conducted of all patients at a single institution who underwent microsurgical OSA clipping with planned TBO. Patient demographics, presentation, aneurysm morphology, occlusion outcomes, complications, recurrence, and functional outcomes based on modified Rankin score (mRS) at follow-up were evaluated. Patients who underwent balloon inflation for proximal control (+ TBO) were compared with those who did not (-TBO).

**Results:**

A total of 34 patients with 35 OSAs were included. A temporary balloon guide catheter was successfully navigated to the cervical carotid in all cases. TBO was performed in 19 patients (20 aneurysms) during aneurysm clipping. Aneurysm sizes ranged from 2.8 to 18.0 mm (mean: 6.7 mm), with neck sizes ranging from 1.6 to 8.1 mm (mean: 4.2 mm). The + TBO group had a significantly higher proportion of wide-necked aneurysms (> 4 mm) compared to the -TBO group (55.0% vs. 26.7%; p = 0.008) and more frequently required anterior clinoidectomy (84.2% vs. 46.7%; p = 0.020). Complete or near-complete (< 2 mm remnant) aneurysm occlusion was achieved in all cases. There was one complication (2.9%) with permanent sequela and median mRS at follow-up was 0. There were no significant differences in complication rates or functional outcomes between the + TBO and -TBO groups.

**Conclusion:**

Endovascular-assisted TBO is a safe and effective minimally invasive alternative to open neck dissection for achieving proximal control during OSA clipping. TBO may be particularly advantageous for managing wide-neck aneurysms.

**Supplementary Information:**

The online version contains supplementary material available at 10.1007/s00701-026-06847-x.

## Introduction

Despite advances in endovascular technology, a subset of patients benefit from open microsurgical clipping of ophthalmic segment aneurysms (OSAs). Microsurgical clipping of OSAs typically requires effective proximal control of the parent internal carotid artery (ICA) for safe and successful clipping [4, 15, 32]. Proximal control minimizes intraoperative blood loss in the event of intraoperative rupture of the aneurysm, reduces aneurysmal turgor to facilitate clip application, and reduces the risk of distal embolization of intraluminal thrombi [32]. However, achieving proximal control in the paraclinoid region can be challenging due to its anatomical complexity. Traditional approaches to achieving proximal control such as exposure of the cervical ICA in the neck through a separate incision, exposure of the petrous ICA in the middle fossa, or local intracranial proximal control of the paraclinoid ICA via dissection beyond the distal dural ring are associated with prolonged operative time and increased surgical morbidity [4, 15, 17].

Temporary balloon occlusion (TBO) via endovascular access presents an innovative alternative method for achieving proximal control during OSA clipping involving the endovascular inflation of an intravascular balloon at the cervical or petrous segment of the ICA [20]. In addition to obviating the need for more invasive surgical interventions to achieve proximal control, TBO also facilitates real-time intraoperative angiography. Proximal ICA control using endovascular balloon assistance has been previously described in only a handful of technical notes and small series [8, 21, 32]. Despite its potential benefits, the safety and efficacy of TBO in this context remain incompletely defined due to limited contemporary outcomes data.

This study evaluates the use of endovascular-assisted TBO as a primary technique for proximal ICA control during microsurgical clipping of OSAs using newer generation endovascular balloon guide catheters. We aim to better define the safety and efficacy of TBO for OSA clipping and evaluate its associated perioperative complication rate and outcomes.

## Methods

### Human ethics and consent to participate

The study complied with institutional guidelines and was approved by the Feinstein Institute for Medical Research Institutional Review Board (IRB #23–0993). Patient consent was waived due to the retrospective nature of the study and review of de-identified data.

### Patient selection and data collection

This retrospective, single-center study was conducted over a 10-year period (2014–2023). Data collected included patient demographics, procedural details (estimated blood loss, surgical time, balloon catheter characteristics, fluoroscopy time, need for anterior clinoidectomy), perioperative complications, and functional and radiographic outcomes based on modified Rankin Scale (mRS) scores at 1-month follow-up and follow-up vessel imaging at 6-months.

Inclusion criteria encompassed adult patients who underwent either elective or urgent microsurgical clipping of OSAs in which TBO was the planned primary method for proximal ICA control. Patients who underwent TBO (+TBO) were compared to those who did not undergo balloon inflation during aneurysm dissection or clipping (-TBO). Although balloons catheters were navigated into the carotid artery, in -TBO cases, the balloon was not inflated due to adequate exposure and primary operator’s decision that proximal control via TBO was not necessary. Any patient who underwent cervical carotid artery exposure in the neck as the primary method of proximal control was excluded. Additionally, patients in whom the OSA was not the target aneurysm but happened to also be treated in the setting of clipping a different target aneurysm(s) were excluded.

Indication for aneurysm treatment included ruptured aneurysms not amenable to primary coiling, progressive neurologic symptoms, young age, family history of aneurysm rupture, evidence of aneurysm growth on imaging, or patient preference. Eligibility for clipping was determined by a multidisciplinary cerebrovascular team and based on progressive symptoms, aneurysm growth, and patient ineligibility for endovascular treatment due to factors such as aneurysm morphology, inability to tolerate long-term antiplatelet therapy, or patient preference. Although endovascular treatment is preferred for most of these aneurysms, this treatment modality has its own risks, including stroke, hemorrhage and mortality. In our contemporary series comparing clipping vs flow diversion in paraclinoid aneurysms, there were two mortalities in the endovascular group.

### Surgical technique

**Fig. 1:**
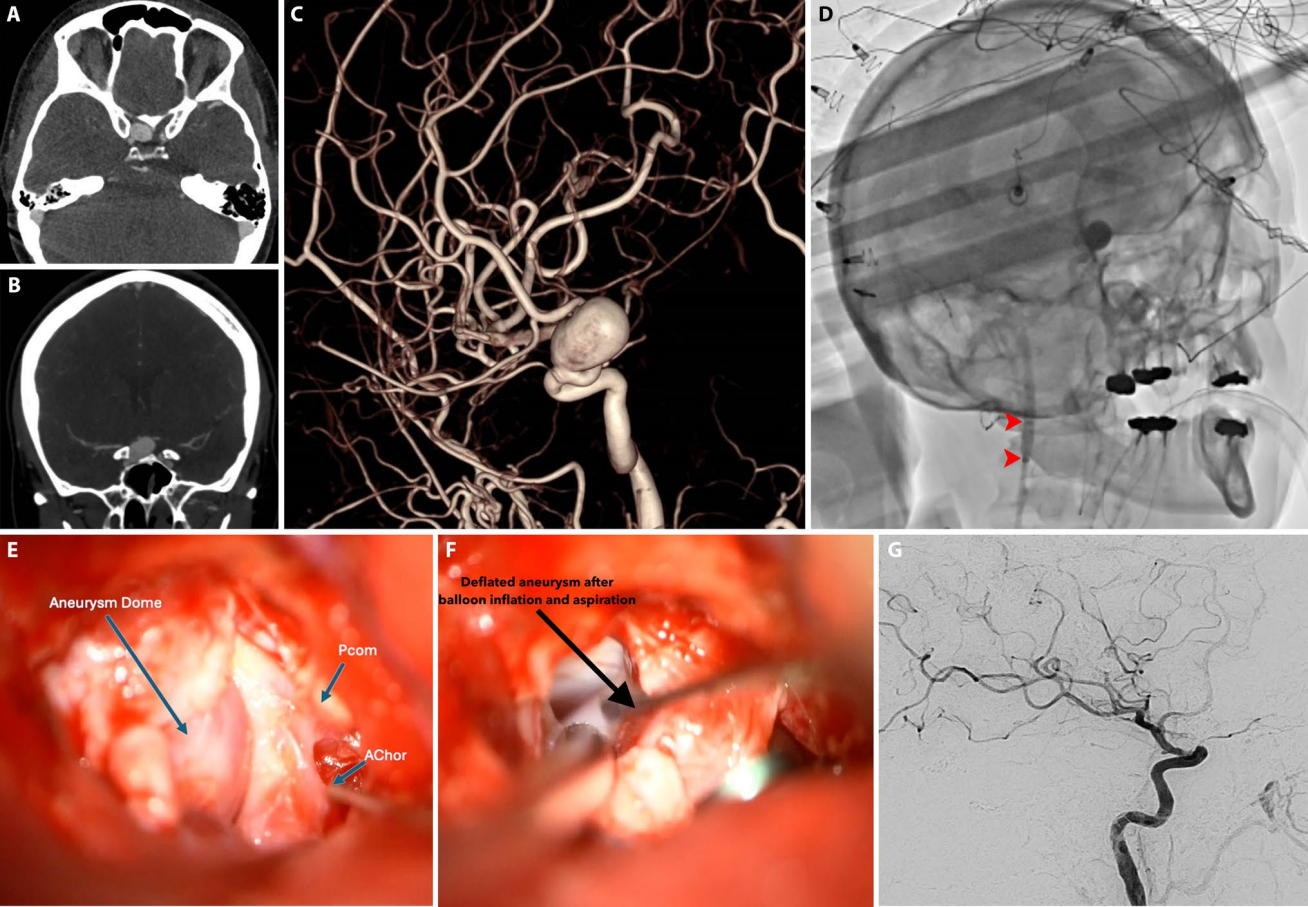
Temporary balloon occlusion (TBO) case example. **A** Axial and **B** coronal CT angiogram showing a large, 16 mm right ophthalmic aneurysm originating proximal to the anterior clinoid process. **C** 3D rotational angiography from a preoperative catheter angiogram right internal carotid artery (ICA) injection showing the large aneurysm in the ophthalmic segment adjacent to the origin of the ophthalmic artery with a 5 mm neck. **D** Intraoperative unsubtracted single plane lateral angiogram, right ICA injection, at the time of balloon inflation (TBO) in the cervical segment of the ICA showing contrast stagnation distal to the balloon (marked proximally and distally by the red arrowheads) and in the aneurysm. **E** Intraoperative exposure after a right pterional craniotomy, intradural anterior clinoidectomy. The origins of the posterior communicating artery (Pcom) and anterior choroidal artery are visible lateral to the large aneurysm dome. **F** Intraoperative view after further dissection and opening of the of the distal dural ring, placement of a temporary clip on the ICA distal to the aneurysm but just proximal to the Pcom, and after inflation of the endovascular balloon (as shown in **D**) with aspiration decompression. The aneurysm has visibly lost its turgor and can be more easily manipulated with microdissectors for precise clip placement. **G** Post-clipping intraoperative lateral angiogram (right ICA injection) demonstrates occlusion of the paraophthalmic aneurysm with persistent patency of the nearby ophthalmic artery

All cases were performed in the operating room with a mobile vascular C-arm or in a hybrid operating suite with single-plane angiography as described below and as exemplified by the case example in Fig. 1. In brief, after general anesthesia induction and sterile preparation, transfemoral arterial access is performed via a modified Seldinger technique. A heparinized saline flush line is then attached to the sheath. An 8-French balloon guide catheter (Cello, Medtronic or Walrus, Q’Apel) or a 6-French guide catheter with a 4 × 20 mm Scepter XC (Microvention, Tustin, CA) is navigated into the cervical and petrous ICA. The balloon and/or guide catheter were also connected to a heparinized saline flush line to ensure continuous heparinization of the catheter (heparin concentration of 2 units/ml at a rate of 30 ml/h). Balloon inflation is then performed under fluoroscopic guidance to determine the volume needed for occlusion, which is marked for future inflations to obviate the need for repeated fluoroscopy. A mini-pterional craniotomy and a retractorless trans-sylvian approach is then used to expose the ICA and optico-carotid cistern. Careful perianeurysmal microdissection is pursued to reveal the OSA. If necessary, an intradural anterior clinoidectomy is performed using a 2 mm diamond drill under constant irrigation. This is determined depending on the location and access to the aneurysm neck for clipping, not necessarily for proximal control. In all cases where anterior clinoidectomy was performed, the distal dural ring was opened. The decision to inflate the balloon during aneurysm dissection and/or clipping is made intraoperatively based on aneurysm size, morphology, and the extent of intracranial ICA exposure that is achieved proximal to the aneurysm neck. Similarly, in cases of large or giant aneurysms, decision to perform aspiration decompression via the balloon catheter was made intraoperatively based on aneurysm size, morphology. This was performed by an assistant operator and using a 60-cc syringe attached to the hub of the guide catheter and aspiration is performed under direct visualization. After clipping, intraoperative angiography is routinely performed both to evaluate aneurysm occlusion and to determine patency of the parent artery and nearby branches. The catheters and balloon guide are removed as soon as the aneurysm is deemed to be successfully treated as to not prolong catheter time and reduce thromboembolic complications [33].

### Statistical analysis

Statistical analysis was performed using Prism v10.3.0 (GraphPad, San Diego, CA). Bivariate analyses were conducted to compare the + TBO and -TBO cohorts using Fisher’s exact test for categorical variables. Nonparametric student’s t-test and Mann–Whitney U tests were used to compare continuous variables, including age, estimated blood loss, and operative time. A value of *p* < 0.05 based on two-tailed hypothesis testing was considered statistically significant.

## Results

### Patient characteristics

A total of 48 patients underwent microsurgical clipping of target OSA aneurysms during the study period. Of these, 34 patients with 35 OSAs had planned TBO as the primary means for proximal ICA control and were included in this study. One (3.0%) patient had two distinct OSA aneurysms on the same side that were clipped during the same procedure. Four (11.8%) of these patients underwent microsurgical clipping in the acute ruptured setting due to inability to safely endovascularly treat the aneurysm without stenting or flow diversion, 4 (11.8%) due to progressive neurologic symptoms, 6 (17.6%) due to large size (> 10 mm), 11 (32.4%) due to patient preference after shared decision making and multidisciplinary discussion, 5 (14.7%) due to presence of another ipsilateral aneurysm which could be treated in the same setting, 4 (11.8%) due to inability to tolerate long-term dual antiplatelet therapy. Nineteen (55.9%) patients underwent TBO during dissection and clipping of their OSA aneurysms (+ TBO). Fifteen (44.1%) patients were prepared for TBO but ultimately did not undergo balloon inflation (-TBO). Intraoperative angiography was performed on all patients after clipping. For 31 cases (91.2%) were performed in a hybrid operating suite. In the remaining three cases, C-arm fluoroscopy was used for the endovascular portion due to the hybrid suite being unavailable.

The majority (88.6%; n = 31) of patients were female with a mean age of 51.1 years (range 26–71). Headache was the most common initial clinical presentation (44.1%; n = 15), followed by incidental findings on neuroimaging (41.2%; n = 14). There were no significant differences in age, clinical presentation, or comorbidities between the + TBO and -TBO groups (Table 1). Hypertension was the most prevalent comorbidity seen in 50.0% of patients. Five (14.7%) patients had additional (non-OSA) aneurysms clipped during the same procedure.

**Table 1:** Patient demographics and presentation

Variable	All *n* = 34 (%)	No TBO *n* = 15 (%)	+ TBO *n* = 19 (%)	*p*-value
Female	30 (88.2%)	13 (86.7)	17 (89.5)	0.999
Mean Age (years) (range)	51.1 (26–71)	53.1 (26–71)	49.6 (29–66)	0.403
Comorbidities				0.942
Diabetes Mellitus	5 (14.7)	3 (20.0)	2 (10.5)	0.634
Hyperlipidemia	8 (23.5)	3 (20.0)	5 (26.3)	0.666
Hypertension	17 (50.0)	6 (40.0)	11 (57.9)	0.491
Smoking History	10 (29.4)	4 (26.7)	6 (31.6)	0.999
Obesity	10 (29.4)	4 (26.7)	6 (31.6)	0.999
Presentation				0.749
Incidental	11 (32.4)	6 (40.0)	5 (26.3)	0.563
Headache	15 (44.1)	6 (40.0)	9 (47.4)	0.609
Seizures	2 (5.9)	1 (6.7)	1 (5.3)	0.999
Neurologic Deficit	2 (5.9)	1 (6.7)	1 (5.3)	0.999
Vision change	4 (11.8)	1 (6.7)	3 (15.8)	0.488
Other aneurysms treated in same setting	5 (14.7)	2 (13.3)	3 (15.8)	0.840

*TBO* Temporary balloon occlusion

### Aneurysm characteristics

Four (11.8%) out of the 35 total aneurysms in the cohort were ruptured on presentation. The majority of OSAs were ophthalmic artery aneurysms (71.4%; *n* = 25) and the remaining were superior hypophyseal artery aneurysms. There were no dorsal carotid wall aneurysms. In terms of aneurysm morphology, largest diameter ranged from 2.9 to 18.0 mm (mean: 7.2 mm). Most aneurysms (82.9%; *n* = 29) were classified as small (< 10 mm). There were no significant differences in dome size between the + TBO and -TBO groups. Neck size ranged from 1.6 to 8.1 mm (mean: 4.2 mm). The majority of aneurysms (54.3%; *n* = 19) had narrow (≤ 4 mm) necks. The + TBO cohort included significantly more aneurysms with wider necks (> 4 mm) than the -TBO cohort (55.0% vs. 26.7%; *p* = 0.008). The aneurysms were predominantly superiorly projecting (60.0%; *n* = 21) with no significant differences in observed projection directions between the + TBO and -TBO groups **(**Table 2**)**.

**Table 2:** Aneurysm characteristics

Variable	All *n* = 35 (%)	No TBO *n* = 15 (%)	+ TBO *n* = 20 (%)	*p*-value
Ruptured	4 (11.4)	3 (20.0)	1 (5.0)	0.299
Aneurysm Size (mm) (range)	6.7 (2.9–18.0)	6.8 (3.5–16)	7.5 (2.9–18.0)	0.626
Small (< 10 mm)	29 (82.9)	13 (86.7)	16 (80.0)	0.605
Large (≥ 10 mm)	6 (17.1)	2 (13.3)	4 (20.0)	0.605
Neck Size				
Narrow (≤ 4 mm)	19 (54.3)	11 (73.3)	8 (45.0)	0.008*
Wide (> 4 mm)	16 (45.7)	4 (26.7)	12 (55.0)	0.008*
Ophthalmic artery aneurysm	25 (71.4)	12 (80.0)	13 (65.0)	0.458
Superior hypophyseal artery aneurysm	10 (28.6)	3 (20.0)	7 (35.0)	0.458
Left Sided	18 (51.4)	9 (60.0)	9 (45.0)	0.499
Projection				0.714
Superior	21 (60.0)	9 (60.0)	12 (60.0)	0.999
Inferior	3 (8.6)	2 (13.3)	1 (5.0)	0.631
Lateral	4 (11.4)	2 (13.3)	2 (10.0)	0.999
Medial	7 (20.0)	2 (13.3)	5 (25.0)	0.672

*TBO* Temporary balloon occlusion, *mm* millimeters; * = indicates statistical significance for α = 0.05

### Operative characteristics and outcomes

Mean estimated blood loss was 163.6 mL (range: 50–300 mL) and mean operative time from induction to extubation was 473.1 min (range 260–720 min) with no differences observed between the + TBO and -TBO cohorts (Table 3). Intradural anterior clinoidectomy was performed in 23 patients (67.6%). A significantly larger portion of patients in the + TBO group underwent anterior clinoidectomy (84.2% vs. 46.7%; *p* = 0.020). Three different intravascular balloons were employed: Cello (44.1%), Walrus (41.2%), and Scepter XC (14.7%). Balloon catheters were used and positioned successfully within the ipsilateral ICA in all cases (+ TBO and -TBO). The balloon types used did not differ between the + TBO and -TBO cohorts. Balloon inflation in the + TBO was technically successful in all cases. The frequency of balloon inflation and deflation varied from 1 to 7 times in a given patient (median: 2). Mean total TBO time was 198.3 s (range 30–840 s) and mean fluoroscopy time was 7.0 min (range 2.4–17.0 min). Two patients additionally underwent aspiration-decompression after balloon inflation due to large aneurysm size to help enable aneurysm manipulation and subsequent clipping. In comparing the four ruptured cases to the unruptured patients, there were no differences between TBO use. The only difference between the cohorts was noted in total fluoroscopy time (11.3 vs. 5.9 min; *p* = 0.037) (Supplemental Table 1).

**Table 3:** Operative variables and outcomes

Variable	All *n* = 34 (%)	-TBO *n* = 15 (%)	+ TBO *n* = 19 (%)	*p*-value
Mean Estimated Blood Loss (mL) (range)	163.6 (50–300)	186.7 (100–300)	154.6 (50–300)	0.162
Mean Operative Time (mins) (range)	473.1 (260–720)	475.2 (280–720)	471.8 (260–570)	0.949
Anterior clinoidectomy	23 (67.6)	7 (46.7)	16 (84.2)	0.020*
Balloon Type				0.210
8 F Walrus	14 (41.2)	5 (33.3)	9 (47.4)	0.409
8 F Cello	15 (44.1)	6 (40.0)	9 (47.4)	0.667
4 × 20 Scepter XC	5 (14.7)	4 (26.7)	1 (5.3)	0.080
Use of aspiration-decompression	2 (5.9)	0 (0.0)	2 (10.5)	0.195
Mean Balloon inflation time (sec) (range)	198.3 (30–840)	-	198.3 (30–840)	-
Mean Fluoroscopy time (mins) (range)	7.0 (2.4–17.0)	7.2 (2.4–17.0)	6.7 (4.8–8.0)	0.785
Intraoperative clip adjustment (n = 35)	10 (28.6)	5/15 (33.3)	5/20 (25.0)	0.589
Complete occlusion of aneurysm (n = 35)	32 (91.4)	14/15 (93.3)	18/20 (90.0)	0.727
Parent artery/branch stenosis (n = 35)	2 (5.7)	1/15 (6.7)	1/20 (5.0)	0.863
All complications	5 (14.7)	3 (20.0)	2 (10.5)	0.439
Asymptomatic Ischemic stroke	2 (5.9)	0 (0.0)	2 (10.5)	0.195
Transient Hemiparesis	1 (2.9)	1 (6.7)	0 (0.0.)	0.253
Vessel Injury	0 (0.0)	0 (0.0)	0 (0.0)	-
Intracerebral Hemorrhage	0 (0.0)	0 (0.0)	0 (0.0)	-
Symptomatic cerebral vasospasm	1 (2.9)	1 (6.7)	0 (0.0)	0.253
Visual deficits	1 (2.9)	1 (6.7)	0 (0.0)	0.253
Median mRS at 1-month (range)	0 (0–2)	0 (0–2)	0 (0–1)	0.390
Radiographic Recurrence	0 (0.0)	0 (0.0)	0 (0.0)	-

*TBO* Temporary balloon occlusion, *mL* milliliters, *mins* minutes, *sec* seconds; * = indicates statistical significance for α = 0.05

Overall, complete aneurysm occlusion was achieved in 32 (91.4%) of the 35 OSA aneurysms, with two near-complete occlusions (< 2 mm remnant) observed in the + TBO cohort and one observed in the -TBO cohort—all left intentionally to help maintain ophthalmic artery patency. There were no intraoperative aneurysm ruptures. Additionally, there were no identified intraoperative vessel injuries (dissection or pseudoaneurysm) caused by balloon inflation. There were two cases of mild to moderate parent artery stenosis (< 30%), based on intraoperative angiogram interpretation. Intraoperative clip adjustment was performed in 10 patients (28.6%) based on intraoperative angiographic findings; there were no significant differences in the need for clip adjustment between the + TBO and -TBO cohorts. One case of parent artery stenosis in the -TBO, required further intervention due to postoperative transient hemiparesis **(**Table 3**)**.

There was one (2.9%) permanent neurologic complication resulting in postoperative ipsilateral quadrantonopsia in a TBO- patient. Two patients (5.9%) experienced transient neurologic complication, both in the -TBO group (Table 3). These included one case of transient contralateral hemiparesis from hypoperfusion necessitating returning to the operating room for clip adjustment and one case of symptomatic vasospasm of the ipsilateral anterior circulation. There were two patients (5.9%) with asymptomatic ischemic strokes seen on imaging, likely related to thromboembolism, both in the + TBO. All patients, except for the one with new visual deficit, had returned to their neurologic baseline prior to discharge with no permanent neurologic sequela. Functional outcome based on mRS ranged from 0–2 at 1-month follow-up and the median mRS was 0 for both the + TBO and -TBO cohorts. The single case with an mRS of 2 at one month follow-up incurred functional disability from that patient’s subarachnoid hemorrhage course **(**Table 3**)**. 29 patients (85.3%) had follow-up vessel imaging demonstrating no aneurysm recurrence or new residual.

## Discussion

This study evaluates the use of TBO for proximal ICA control during microsurgical clipping of OSAs and highlights its feasibility, safety, and clinical utility. Proximal vessel control during clip application is well-established during microsurgical intervention for aneurysms [31]. Traditional techniques including parent vessel temporary occlusion via a secondary surgical access have been associated with increased surgical time and morbidity [4, 32]. Due to the anatomical complexity of the paraclinoid region, achieving proximal control intracranially can be particularly challenging. This study presents a contemporary series of OSAs treated via microsurgical clipping and endovascular-assisted use of newer generation balloon guide catheters that have not been described in prior studies. Additionally, our study is the first to directly compare patients who underwent balloon inflation versus those that did not. Our results suggest that TBO is a reliable alternative to conventional methods for achieving proximal control, particularly for OSA aneurysms with wide-neck morphology or those requiring anterior clinoidectomy for sufficient exposure.

Despite advances in endovascular techniques for managing aneurysms, microsurgical clipping is not obsolete and remains an essential treatment option that continues to demonstrate superior long-term angiographic outcomes in randomized trials [5, 6, 33]. At the same time, growing familiarity with endovascular techniques has expanded the role of adjunctive methods such as TBO. Prior studies, largely limited to case reports and small series, have described TBO during clipping of paraclinoid [2, 3, 8, 9, 11, 13, 14, 20–23, 25–30, 32, 34, 35] and posterior circulation aneurysms [18, 28]. However, these studies describe older generation balloon guides and focus on techniques for large and giant paraclinoid aneurysms, especially in conjunction with retrograde suction-decompression [2, 3, 9–11, 14, 20, 21, 23, 26, 30, 34]. This combination technique was successfully used in two patients with large aneurysms (18 and 16 mm) in our cohort. Of note, balloon guide catheters are required to perform aspiration-decompression adequately given the limitation of balloon microcatheters due to their significantly smaller inner diameter. Therefore, our preference has been to use balloon guide catheters (eg. Walrus, Q’Apel) in cases of large or giant aneurysms.

Previous studies have suggested that the addition of TBO does not significantly increase procedural risk. For example, the largest—albeit older—study, Fulkerson et al., analyzed 63 ophthalmic artery aneurysms and found comparable complication rates between cases with and without TBO and suction decompression [11]. Other investigators have also noted an additional advantage of TBO as a potential salvage technique to promote hemostasis and preserve the parent vessel during clip placement [8]. Additionally, TBO has been suggested to decrease surgical time and reduce the need for brain retraction by providing proximal vessel control prior to any initial aneurysm manipulation or dissection [18]. In terms, of radiation time, Zhang et al. refined the technique with the use of methylene blue into the balloon, to improve visibility under the microscope, optimize its positioning, and avoid excess radiation exposure [35].

In our study, complete aneurysm occlusion was achieved in 91.4% of cases, consistent with the existing literature on microsurgical and endovascular treatment of OSAs [1, 21]. Near-complete occlusion (< 2 mm remnant) was observed in three cases, in which the small remnant was left intentionally to preserve the parent ophthalmic artery. While the rate of complete occlusion was not different between the + TBO and -TBO groups, we attribute the overall high occlusion rate to the utility of TBO in facilitating safer dissection and manipulation of the aneurysm and making clip placement more precise in large or wide-necked aneurysms [21], especially when used in conjunction with aspiration-decompression.

Despite its advantages, TBO introduces some potential risks, such as vascular injury, vessel dissection, vasospasm, and thromboembolic complications [19]. During craniotomy, the absence of systemic heparinization—commonly used in balloon test occlusion or balloon-assisted coiling—creates additional thromboembolic risk. Furthermore, repeated balloon inflations may increase this risk without adequate anticoagulation [27]. A review of prior studies indicates that TBO inflation durations typically range from 1.5 to 3 min per inflation, with a maximum total occlusion time of 50 min. No clear association has been demonstrated between balloon inflation duration and post-procedural complications [18]. In our cohort, individual inflation times were not recorded, but total occlusion time ranged from 0.5 to 14 min. Reported TBO-related complication rates, including thromboembolic events, vasospasm, and ICA dissection, range from 1.7% to 3.7% [2, 18]. In this study, the observed complications could not be definitively attributed to TBO. Our findings are similar to those in Fulkerson et al., which cited a complication rate of 17.6% in the ruptured aneurysm subgroup and a 9.5% overall complication rate for patients undergoing TBO [11]. Only one patient in our series developed a permanent deficit (postoperative quadrantanopia), while all other ischemic events did not result in lasting neurological sequelae.

Additionally, no cases of branch artery compromise were observed and only two patients (5.9%) developed mild or moderate stenosis of the parent ICA due to clip impingement. One patient who underwent clip reconstruction with six clips for a 16-mm ophthalmic artery aneurysm exhibited symptoms of ipsilateral hemispheric hypoperfusion postoperatively. The patient required reoperation, during which the aneurysm and ICA were re-explored and one of the clips was readjusted, which subsequently resulted in resolution of the hypoperfusion symptoms.

Thromboembolic events, vessel perforation and dissection are known complications of balloon-assisted embolization techniques [1, 19]. Vessel wall injury and thromboembolism are two of the most commonly reported complications associated and may occur at a rate as high as 7.5% [1, 19, 24]. In our series, two patients in the + TBO group experienced ischemic complications that could possibly be related to the balloon catheter. In both patients, the balloon was inflated more than once. One patient, who had a history of sickle cell disease, developed an intraoperative acute thrombus due to inadvertent interruption of the heparinized saline flush but fortunately did not experience any long-term clinical consequences. However, this highlights the need for careful monitoring of the heparinized line during the procedure. In the second case, the patient’s postoperative CT imaging showed an asymptomatic ipsilateral parieto-occipital hypodensity, likely an infarction from thromboembolic event. In the other case, balloon inflation time exceeded 10 min, and fluoroscopy time was also high (10.1 min), reflecting possible difficulty in positioning the catheter. In cases where a thromboembolic event leads to a large or medium vessel occlusion, management in an operating room can be more challenging than in a biplane angiography suite due to relative resource limitations, the potential need to navigating large catheters intracranially across an aneurysm for potential thrombectomy, and the significant risks associated with systemic anticoagulation during craniotomy. Given the iatrogenic risk, better patient selection based on aneurysm neck size and location in relation to the anterior clinoid can be performed to avoid unnecessary use of balloon catheters.

In our series, anterior clinoidectomy was performed in 67.6% of cases and significantly more frequently in the + TBO group than in the -TBO group (84.2% vs. 46.7%; *p* = 0.020). In these cases, the clinoidectomy was performed primarily to achieve adequate exposure of the aneurysm neck for clipping, rather than for proximal control, which was instead achieved through TBO. This explains the substantial proportion of patients who underwent both TBO and anterior clinoidectomy. It also suggests that when sufficient visualization of the proximal aneurysm neck could be achieved without clinoidectomy, the use of TBO was less likely to be necessary. Given the regional anatomy, the optic nerve is particularly vulnerable during OSA clipping, with postoperative visual deficits reported in up to 28.5% of cases​​ [16]. Anterior clinoidectomy poses additional risks, including direct optic nerve manipulation, vibrational injury from drilling, ischemia, and optic nerve compression from the clip itself [7, 16]. Notably, only one patient developed a postoperative ipsilateral quadrantanopia, which occurred in a patient in the -TBO cohort who did undergo an intradural clinoidectomy (although the exact etiology remains unclear despite consideration of an ischemic cause vs. clinoidectomy vs. optic nerve manipulation).

In summary, despite advances in flow diversion for these aneurysms, select group of patients remain to benefit from microsurgical clipping of OSA aneurysm when indicated and other treatment modalities are not possible. In these patients, TBO provides a safe and effective method for proximal ICA control during microsurgical OSA clipping as an alternative to cervical carotid exposure. Our findings suggest that TBO is particularly advantageous for wide-neck aneurysms and in cases requiring anterior clinoidectomy. Potential complications, such as thrombus formation and vasospasm, appear to be infrequent and may be mitigated with meticulous technique, heparinized saline flushes, and vasodilators. The minimally invasive nature of TBO aligns with the growing trend toward hybrid surgical-endovascular approaches in cerebrovascular surgery​ [12, 23]. Future advancements in balloon catheter design, such as more compliant balloons, will likely enhance procedural precision and safety.

### Limitations

This study is limited by its small sample size, single-center setting, and retrospective design, all of which can impact generalizability. Additionally, surgical outcomes and complication rates may vary across institutions depending on surgeon and endovascular team expertise. MRI was not routinely performed, and smaller asymptomatic infarcts could have been missed. Furthermore, variability in aneurysm morphology and surgical complexity within our cohort makes it difficult to identify subgroups that would benefit most from TBO. Selection bias as to who underwent TBO versus did not is also a limitation as more complex cases may have undergone TBO. Despite these limitations, our findings support the goal of our study which is to evaluate the utility of TBO for proximal ICA control in OSA clipping. A comparison between TBO use versus cervical exposure would have provided additional information regarding the benefits of our endovascular-assisted approach for proximal exposure. Practically, this technique requires the use of a hybrid operating suite or specific radiolucent head-holders and a C-arm which may increase costs or not be readily available in some countries, limiting its applicability. Future multicenter studies with larger cohorts are warranted to validate these findings and refine patient selection criteria. Additionally, the integration of advanced imaging techniques, such as 3D rotational angiography, could further optimize preoperative planning and intraoperative decision-making regarding the need for TBO.

## Conclusion

TBO is a valuable adjunct during microsurgical clipping of OSAs as it can provide effective proximal ICA control with a favorable safety profile and outcomes. The minimally invasive nature of TBO can be used as an alternative to proximal carotid exposure which involves additional surgical exposure or secondary incisions. A high aneurysm occlusion rate was observed in this series with endovascular-assisted TBO, and TBO was found to be particularly efficacious in cases involving wide-necked aneurysms or aneurysms requiring anterior clinoidectomy for optimal exposure. As both endovascular and microsurgical techniques continue to evolve, TBO could play an increasingly integral role in the multidisciplinary management of intracranial aneurysms.

## Supplementary Information

Below is the link to the electronic supplementary material.

Supplementary file1 (DOCX 23 KB)

## Data Availability

No datasets were generated or analysed during the current study.
