# Supplementary material for: Endovascular-assisted microsurgical clipping of ophthalmic segment aneurysms
Source: Acta Neurochir (Wien). 2026 Mar 26;168(1):87. doi: 10.1007/s00701-026-06847-x (PMC13031223; doi:10.1007/s00701-026-06847-x)
Supplement: Supplementary file 1 — Supplementary file1 (DOCX 23 KB) [file 701_2026_6847_MOESM1_ESM.docx]

**`Supplemental Table 1.** Comparison of ruptured and unruptured OSA aneurysm cases.

| **Variable** | **All**  **n=34 (%)** | **Ruptured**  **n=4 (%)** | **Unruptured**  **n=31 (%)** | **p-value** |
| --- | --- | --- | --- | --- |
| Female | 30 (88.2%) | 3 (75.0) | 27 (87.1) | 0.476 |
| Mean Age (years) (range) | 51.1 (26-71) | 47.3 (29-56) | 51.5 (26-71) | 0.428 |
| Median Hunt Hess Grade | NA | 2 (2-3) | NA | - |
| Presentation |  |  |  |  |
| Incidental | 11 (32.4) | 0 (0.0) | 11 (35.5) | 0.284 |
| Headache | 15 (44.1) | 2 (50.0) | 13 (29.0) | 0.999 |
| Seizures | 2 (5.9) | 1 (25.0) | 1 (3.2) | 0.218 |
| Neurologic Deficit | 2 (5.9) | 1 (25.0) | 1 (3.2) | 0.218 |
| Vision change | 4 (11.8) | 0 (0.0) | 4 (12.9) | 0.999 |
| Other aneurysms treated in same setting | 5 (14.7) | 1 (25.0) | 4 (12.9) | 0.476 |
| Aneurysm Size (mm) (range) | 6.6 (2.9-18.0) | 5.5 (2.9-7.0) | 7.7 (4.1-18.0) | 0.401 |
| Small (<10mm) | 4 (82.9) | 4 (100.0) | 25 (80.6) | 0.999 |
| Large (≥10mm) | 6 (17.1) | 0 (0.0) | 6 (19.4) | 0.999 |
| Neck Size |  |  |  |  |
| Narrow (≤4mm) | 19 (54.3) | 3 (75.0) | 16 (51.6) | 0.608 |
| Wide (>4mm) | 16 (45.7) | 1 (25.0) | 15 (48.4) | 0.608 |
| Ophthalmic artery aneurysm | 25 (71.4) | 4 (100.0) | 21 (67.7) | 0.302 |
| Superior hypophyseal artery aneurysm | 10 (28.6) | 0 (0.0) | 10 (32.3) | 0.302 |
| Left Sided | 18 (51.4) | 3 (75.0) | 15 (48.4) | 0.602 |
| Projection |  |  |  |  |
| Superior | 21 (60.0) | 3 (75.0) | 18 (58.1) | 0.635 |
| Inferior | 3 (8.6) | 0 (0.0) | 3 (9.7) | 0.999 |
| Lateral | 4 (11.4) | 1 (25.0) | 3 (9.7) | 0.399 |
| Medial | 7 (20.0) | 0 (0.0) | 7 (22.6) | 0.562 |
| Anterior clinoidectomy | 23 (67.6) | 3 (75.0) | 20 (64.5) | 0.999 |
| TBO | 19 (55.9) | 2 (50.0) | 17 (54.8) | 0.999 |
| Balloon Type |  |  |  |  |
| 8F Walrus | 14 (41.2) | 0 (0.0) | 14 (45.2) | 0.133 |
| 8F Cello | 15 (44.1) | 2 (50.0) | 13 (41.9) | 0.999 |
| 4x20 Scepter XC | 5 (14.7) | 2 (50.0) | 3 (9.7) | 0.088 |
| Mean Balloon inflation time (sec) (range) | 198.3 (30-840) | 480.0 (120-840) | 162.3 (30-600) | 0.094 |
| Mean Fluoroscopy time (mins) (range) | 7.0 (2.4-17.0) | 11.3 (7.8-17.0) | 5.9 (4.8-7.2) | 0.037* |
| Intraoperative clip adjustment (n=35) | 10 (28.6) | 1 (25.0) | 9 (29.0) | 0.999 |
| Complete occlusion of aneurysm (n=35) | 32 (91.4) | 4 (100.0) | 28 (90.3) | 0.999 |
| Parent artery/branch stenosis (n=35) | 2 (5.7) | 1 (25.0) | 1 (3.2) | 0.218 |
| All complications | 5 (14.7) | 1 (25.0) | 4 (12.9) | 0.476 |
| Asymptomatic Ischemic stroke | 0 () | 0 (0.0) | 2 (6.5) | 0.999 |
| Transient Hemiparesis | 1 (2.9) | 1 (25.0) | 0 (0.0) | 0.114 |
| Vessel Injury | 0 (0.0) | 0 (0.0) | 0 (0.0) | - |
| Intracerebral Hemorrhage | 0 (0.0) | 0 (0.0) | 0 (0.0) | - |
| Symptomatic cerebral vasospasm | 1 (2.9) | 0 (0.0) | 1 (3.2) | 0.999 |
| Visual deficits | 1 (2.9) | 0 (0.0) | 1 (3.2) | 0.999 |
| Median mRS at 1-month (range) | 0 (0-2) | 1 (0-2) | 0 (0-1) | 0.596 |

TBO=temporary balloon occlusion; mL=milliliters; mins=minutes; sec=seconds; *=indicates statistical significance for α=0.05.
